# High-throughput microbead assay system with a portable, cost-effective Wi-Fi imaging module, and disposable multi-layered microfluidic cartridges for virus and microparticle detection, and tracking

**DOI:** 10.21203/rs.3.rs-2383455/v1

**Published:** 2022-12-20

**Authors:** Jorge Manrique Castro, Frank Sommerhage, Rishika Khanna, Andre Childs, David DeRoo, Swaminathan Rajaraman

**Affiliations:** University of Central Florida; Primordia Biosystems; Indian Institute of Technology Delhi; University of Central Florida; Primordia Biosystems; University of Central Florida

**Keywords:** Microfluidic system, Virus detection, Microparticle tracking, Multilayered chip, High-troughtput assay

## Abstract

In recent years biomedical scientific community has been working towards the development of high-throughput devices that allow a reliable, rapid and parallel detection of several strains of virus or microparticles simultaneously. One of the complexities of this problem lies on the rapid prototyping of new devices and wireless rapid detection of small particles and virus alike. By reducing the complexity of microfluidics microfabrication and using economic materials along with makerspace tools (Avra Kundu, Ausaf, and Rajaraman 2018) it is possible to provide an affordable solution to both the problems of high-throughput devices and detection technologies. We present the development of a wireless, standalone device and disposable microfluidics chips that rapidly generate parallel readouts for selected, possible virus variants from a nasal or saliva sample, based on motorized and non-motorized microbeads detection, and imaging processing of the motion tracks of these beads in micrometers. Microbeads and SARS-CoV-2 COVID-19 Delta variant were tested as proof-of-concept for testing the microfluidic cartridges and wireless imaging module. The Microbead Assay (MA) system kit consists of a WiFi readout module, a microfluidic chip, and a sample collection/processing sub-system. Here, we focus on the fabrication and characterization of the microfluidic chip to multiplex various micrometer-sized beads for economic, disposable, and simultaneous detection of up to six different viruses, microparticles or variants in a single test, and data collection using a commercially available, WiFi-capable, and camera integrated device (Fig. 1).

## 1. Introduction

Microfluidics platforms (Kovacs 1998) are being developed and deployed all around us in a variety of ways, starting from single purpose, disposable chips to analyze specific biological samples (Azim et al. 2018), integrated into a broader system as the initially proposed Micro Total Analysis System (μTAS) (Manz, Graber, and Widmer 1990) for running chemistry on a chip and sample manipulation (Harrison et al. 1992), or into fully microfluidically integrated on-chip biosensors for organs modeling as demonstrated recently by Ding et al (Ding, Zhang, and Wang 2021).

These microfluidics platforms for biomedical applications are moving away from traditional soft lithographic techniques such as Poly(dimethylsiloxane) (PDMS) micromolding (Gokaltun et al. 2017; Trantidou et al. 2017), or glass laser micromachining (Butkutė et al. 2021; Ren, Ganapathysubramanian, and Sundararajan 2011), and instead using other novel and cost effective approaches. For instance, 3D printing based microfluidic chips have been deployed towards bacterial detection (A. Kundu et al. 2019), and protein purification (Habib et al. 2022). Laser micromachining on rapid-prototyping has proven to be a successful technology to process polymers on microstructures and microfluidics applications (Raciukaitis and Gedvilas 2005; Matellan and del Río Hernández 2018). Optimization of parameters for laser ablation, engraving and micro/nanomachining is possible at high precision on intricated features (Ravi-Kumar et al. 2019; Sahu, Malhotra, and Jha 2022). In applications where passive flow channels are required, surface roughness treatments are necessary to control the wettability of the materials forming microchannels (Quéré 2008; Zhong, Wang, and Zirajutheen 2005). Plasma treatments with Oxygen (O2) for PDMS (Tan et al. 2010), and (O2/CF4) for Polymethyl Methacrylate (PMMA) (Sui et al. 2021), have been investigated showing good results for reducing the contact angle and as a result increasing hydrophilicity. In a similar manner, depending on the type of liquid flow (e.g. oil- or water-based), and microfluidic application such as droplet generation (Angely 2020), some treatments can be designed to increase hydrophobicity (Su et al. 2019; Ogończyk, Jankowski, and Garstecki 2020).

Lately, double-sided adhesives (DSA) based microfluidic fabrication strategies are being developed for multilayered microphysiological systems and cellular diagnostics (Hosic et al. 2021; Lucas et al. 2022). These newer microfabrication strategies are being implemented using novel designs, appropriate selection of materials, and operating at the limits of common microfabrication technologies. Laser micromachining of DSA is an interesting approach for the fabrication of biomedical devices as it allows the creation of hybrid structures using polymers, thermoplastics, elastomers, glass, silicon, hydrogels, resins and other transparent/translucent substrates for optical readout (Zamora et al. 2017; Tsao and Syu 2020; Cooksey and Atencia 2014). One recent review article that illustrates this expansion is presented by Ingber (Ingber 2022), detailing the approaches for the expansion of microfluidics into more complex platforms such as human organs-on-chips and personalized medicine that allow drug development and disease modeling more efficiently. Integration of techniques such as impedance electrochemical spectroscopy on the previously mentioned systems provides additional information on the analysis of specific peripheral nerve studies (Avra Kundu et al. 2021), and understanding of vascular processes (e.g. hemostasis) in humans (Karimi et al. 2019).

Microfluidics are additionally playing a role in addressing challenges caused by recent global health events. With the advent of the coronavirus disease (COVID-19) and its rapid propagation around the world with multiple, and highly contagious variants, clinical testing of individuals remains not dynamic enough to prevent the spread of rapidly evolving viral targets such as SARS-CoV-2 (Chen et al. 2020; Mandal, Dutta, and Dutta 2021). Several tests being used in the laboratory, in-home, and in point-of-care (POC) setting (Harpaldas et al. 2021) have been deployed to combat the pandemic (Safiabadi Tali Seyed et al.). In contrast to most state of the art tests involving microscopy and off-chip assays (Fuchs et al. 2021), microbeads assays such as the Rolosense Assay (RA) (Yehl et al. 2016; Bazrafshan et al. 2021) is a label-free assay that does not require complex fluorescence (Bucevičius et al. 2020), colorimetric and luminescence read-outs (Paterson et al. 2017), nor does the biological sample require exhaustive reverse transcription and DNA amplification as in Polymerase Chain Reaction (PCR) testing. Similar label-free approaches have been proposed, for example: antibody-functionalized gold nanoparticles and enhanced surface plasmon resonance (SPR) techniques were reported as a method to detect SARS-CoV-2 nucleocapsid protein, but specialized tools such as a SPR system and scanning electron microscope (SEM) are required for data analysis (Yano et al. 2022). Another study proposed a reagent free platform using an antibody-based microwave sensor working as a split ring resonator. The shift in the resonant frequency of the resonator upon antigen binding is measured, but this sensor requires further optimization on the readout system as its proof of concept was based on bulky instrumentation like a vector network analyzer for frequency measurements, and a syringe pump system to flow the sample along the microfluidic sensing area (Cui et al. 2022).

To test the viability of the MA system, a proof-of-concept virus detection experiment is showcased under the RA protocol. Single purpose test requires a nasal swab or saliva sample (reagents and sample pre-mixed) inserted into POC detection platform (pre-functionalized cartridge) for whole virion detection via DNA motors coated with virus binding ligands. The motors are 5 m spheres which exhibit motion in the absence of virus and stall in the presence of virus. The mechanism of movement is based on the “burnt bridge” Brownian ratchet evidencing forces in the pN scale (Antal and Krapivsky 2005; Blanchard et al. 2019). DNA-coated particle hybridizes on the RNA coated substrate, and upon addition of RNase H as the chemical fuel, it rolls upon hydrolysis creating depletion tracks on the substrate. Microfluidics can increase the throughput of the sample collection on chip to run parallel testing. A portable reader with reliable optical readout and wireless functionality that can be reused with consumable, disposable microfluidic chips is necessary for POC and in-home application.

In this work, we propose a fast, in-home/POC solution for high-throughput and low cost MA utilizing a Bis[3 (trimethoxysilyl)propyl]amine (Bis-TPA) surface treated (Jang, Park, and Lee 2014), multi-layered microfluidics chip to detect multiple different viruses and/or different variants of viruses (e.g., original COVID-19, Delta, or Omicron variants) in parallel without electrical crosstalk (Fu et al. 2020), which is eliminated due to optical detection principle. This assay optimization follows a simple multi-step procedure: sample collection (nasal swab), incubation (cartridge ports), sample loading and flowing (microchannels), and readout (detection kit + mobile phone), all performed in approximately 15 minutes.

## 2. Materials And Methods

### 2.1 Microfluidic chip microfabrication

Disposable microfluidic cartridges were designed using CAD software (Solidworks, Dassault Systèmes) in a multi-layered fashion as presented schematically in Fig. S-4. The top layer corresponding to the ports, was implemented processing two materials: Polycarbonate (PC) sheet (75×25×1.5 mm TUFFAK), and Polymethyl Methacrylate (PMMA) sheet (75×25×3 mm, Glowforge). The middle layer corresponding to the microchannels of 1.5 mm length, was created using double side adhesive (DSA) tape from two manufacturers (96042, 3M; and RTS3851-17, MEDCO respectively). The mechanical structure for both sides of the DSA tape is liner/silicone adhesive/Polyethylene Terephthalate (PET) carrier/silicone adhesive/liner. Lastly, the bottom layer corresponding to the substrate was created using metallization of thin coverslips 25×75 mm and 170 μm +/− 5 μm thick (#1.5H D 263 M Schott glass, Ibidi USA).

#### 2.1.1 Metallization

Glass substrate (Coverslips unsterile, Ibidi Inc., USA) for the bottom layer was cleaned using standardized protocol with soap, deionized (DI) water, isopropyl alcohol (IPA), acetone, and further drying with nitrogen gun. Metallization was carried out using a E-beam evaporator tool (FC-2000, Temescal) at 4×10^−6^ Torr pressure. First, chromium at 3 nm, and then gold at 5 nm were deposited onto the glass coverslips at a rate of 0.1 Å/s each, and 1% and 9% power (10 kW source) respectively.

#### 2.1.2 Micromilling

PC material was micromachined using a specialized tabletop milling tool (Quick Circuit J5, T-Tech). Two types of bits were used (DB-0960 2.3 mm drill bit for the ports, and Mill-T2 milling bit for the edges). The tool was set at 60,000 RPM and 2.5 mm depth of cut for drilling, and 25 mm/s speed and 2 passes for milling. Also, DSA layer was micromachined with the same tool for generation of microchannels (width: 681, 570, 312, and 237 mm) using the in-house “Air-Gap Micromilling” approach (i.e., providing a gap between the material being cut and the bottom of the stage to avoid bit breaking) that allowed us to use drilling bits of different sizes (0.635, 0.5, 0.25, and 0.2 mm diameter) keeping a safe working distance to achieve microchannels of varying widths.

#### 2.1.3 Cutter plotter

DSA was additionally micromachined using precision plotter cutting (Cameo 4, Silhouette) to generate microchannels and ports features from the CAD design (microchannel widths ranging from 100 up to 600 μm, and port diameters ranging from 1400 up to 4000 μm). The material selected on the tool software was double side adhesive, and the setting for parameters were as follows: Force: 33, Speed: 3, Depth: 4, and Passes: 2. Microchannels of diameter were tested from 100 to 600 μm with increases of 100 μm in each iterative design.

#### 2.1.4 Laser

CO_2_ class 4 laser (Glowforge PRO, Glowforge) served as tool for micromachining both DSA and PMMA. The tool operated in continuous mode at 10,600 nm and its maximum power is rated at 45 watts. For micromachining the DSA, the tool was set at 200 speed and 16% power. For cutting the PMMA material, the tool was set to automatic mode. Double side adhesive layer multimodal laser characterization (Hart and Rajaraman 2020) was carried out using a Class IIIB QuikLaze 50ST2 multimodal laser (New Wave Research Inc., Fremont, CA, USA) using IR (1064 nm), Green (532 nm) and UV (355 nm) modes of micromachining and a maximum pulse energy of 5 mJ/pulse. Circles and lines features were tested while varying process parameters such as power, scan speed, repetition rate, and number of passes as presented in Table S-1.

#### 2.1.5 Bonding

All the layers of the microfluidic chip were bonded in a serial fashion (layer by layer) using a 3D printed (Clear v4 resin, Form 3 printer, Formlabs) support base structure of the same dimensions as that of the glass coverslips to hold the pieces for hand assembly as presented in the optical images in Fig. S-5.

### 2.2 Wi-Fi optical imaging kit microfabrication

The imaging kit was fabricated using the integration of easy-to-find items described below, using commercially available electronics and optical components. The assembled system cartridge was fabricated using additive manufacturing, and in-house scripts developed in Octave and Python allowed for the tracking of microparticles.

#### 2.2.1 ESP-32 CAM module

This module represents a cost-effective developing board with integrated camera, two high performance 32-bit CPUs, Wi-Fi capability, traditional and low power Bluetooth. From images of 1600×1200 pixel size resolution, the module provided 2.2 μm/pixel resolution for particle detection. This ESP32-CAM module was manufactured by Ai-Thinker (Xixiang, China) and can be purchased from most major electronics outlets.

#### 2.2.2 Optical system and accessories

Supporting the optical system (ESP-32 CAM), a dermatological lens (25x, from Supereyes, Shenzhen, China) was implemented into the kit with two accompanying white LilyPad LEDs on each side (SparkFun Electronics, CO, USA). This improved the image quality and particle tracking into a field of view of 12mm x 9mm.

#### 2.2.3 Image processing software analysis

Object detection and stalling was processed at a rate of three images per minute (seen as darkfield-like microscopy). The script was developed using Octave and Python with the data being stored in a 8GB internal memory. Removal of invalid tracking, track smoothing and drift compensation techniques were programmed into the script.

#### 2.2.4 Wi-Fi, web server, and user interface development

Accessibility to virus detection and particle tracking plot is obtained wirelessly by connecting any smartphone, laptop, or tablet with Wi-Fi connectivity to the Soft-AP (access point) developed into the ESP-32 CAM module. Configuration of the Soft-AP, firmware update settings and other optical settings programmed into the system can be observed in the web interface as presented in the optical image, Fig. S-6.

### 2.3 Protocol, system and components characterization, and modelling

#### 2.3.1 Microbead detection and tracking

Proof-of-concept experiments involving the microfluidic chips for detection and tracking of virus functionalized microbeads was performed through a chemical assay using COVID-19 Delta variant detection. The selected protocol was the RA protocol. The Rolosenseä Assay was run on the microfluidic cartridges developed in this research, and the resultant data was analyzed using two separate optical microscopy approaches: customized Wi-Fi imaging module as described above, and Eclipse Ti2-E Nikon Inverted Research Microscope using the Elements software package (Nikon). Actual coronavirus, COVID-19 Delta variant (Emory virus bank) was used for virus detection on fluorescence and dark-field imaging setups, and microparticle tracking and detection on the ESP-32 CAM module.

For microparticle analysis, Polystyrene (79633-5ML-F, 55463-5ML-F, Sigma Aldrich) and Silica (SA06N, Bangs Laboratory) beads of 5 μm and 10 μm diameter were mixed with DI water at concentrations of 10 μL/mL and 20 μL/mL. The solution was subsequently flowed along the microchannels from 50 μL/min up to 25 mL/min using an automatic pumping system (Model 200, KDScientific) with intermediate values of 1000 μL/min, 2000 μL/min, 5 mL/min, and 10 mL/min.

#### 2.3.2 Microfluidic cartridge characterization

Laser confocal microscope (VK3050, Keyence) was used to obtain surface roughness measurements using the laser source (Red 661 nm), and focus variation method for 3D profiles, and 2D features measurements of the channels and ports on the microfluidic cartridges.

Effects of the Bis[3 (trimethoxysilyl)propyl]amine, (technical grade, ≥90% Bis-TPA 413356-50ML, Sigma Aldrich) reagent on the microfluidic chips was characterized by UV-Visible spectroscopy, and Contact Angle Measurements (CAM). UV-Visible spectroscopy was performed using an Evolution 220 (Thermo Scientific) from 200 to 1000 nm spectra range on clear PMMA, PC substrates and using glass substrates as references for each corresponding set. Both different concentrations of the Bis-TPA treatments for surface functionalization (i.e., 0.1, 1, and 2% V/V with Ethanol), and the method of overnight drying (ambient and oven) were evaluated. Lastly, CAM was obtained using a Dataphysics automatic goniometer (DataPhysics Instruments USA Corp) dispensing 2 μL and 3 μL water drops onto control, plasma treated (O2 Plasma@40 cc/min for 100 seconds), and Bis-TPA functionalized (0.1%, 1%, and 2% V/V concentration with Ethanol) samples. Both, standard Right-Left (RL) angle and Young-Laplace (YL) measurements were evaluated.

#### 2.3.3 Fluid dynamics modelling

Pressure and shear stress modelling were conducted using Solidworks CAD software package with the Flow Simulation Add-in (Solidworks, Dassault Systèmes) at the lower and upper limits of the experimental flow rates (i.e., 50 μL/min, and 25 mL/min).

All the collected data for the different characterizations utilized in this paper were post-processed using Origin software (OriginLab) for statistical analysis and standardized plots.

## 3. Results And Discussion

### 3.1 Fully integrated Microbead Assay (MA) system

**Figure 2:**
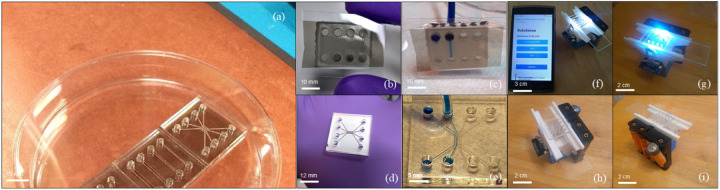
(a) High-throughput disposable PMMA microfluidic cartridge. (b-c) 4-CH Polycarbonate (PC) microfluidic cartridge under leaking and flowing testing. (d-e) 4-CH device on a reduced field of view (2.5 × 3.5 mm) design under leaking and flowing testing. (f-i) Actual kit operating on Wi-Fi connectivity paired to a cell phone showing the graphical user interface (GUI), and 4x AA batteries allowing off-grid powered operation (6 VDC)

The physical structure of the assay system is composed by two essential components: Wi-Fi imaging module and microfluidic chip as depicted in Fig. 2. Integration of the optical system (ESP-32 CAM module, lenses, LEDs) within a 3D printed housing was fabricated in such a way that customized microfluidic chips are placed manually by the user into the system, and external power supply option is available through AA batteries. Additionally, a prototype mobile app is depicted in the lead author’s cell phone in Fig. 2. The overall footprint of the system was: L=5.5 cm, W=4.5 cm, and H=4.5 cm. This serves as a suitable interface for home-deliverable, disposable systems similar to Lateral Flow Assay (LFA) devices already available for COVID-19 detection (Grant et al. 2021; Kivrane et al. 2022; Ragnesola et al. 2020).

To microfabricate the most suitable microfluidic chip compatible with MA, three different micromachining technologies were developed and optimized: micromilling, automatic craft cutting, and laser ablation. Laser confocal microscopy characterization was performed to identify metrics related to the design of the chip and the measured device dimensions and surface roughness as described in the following sections.

### 3.2 Physical characterization

**Figure 3:**
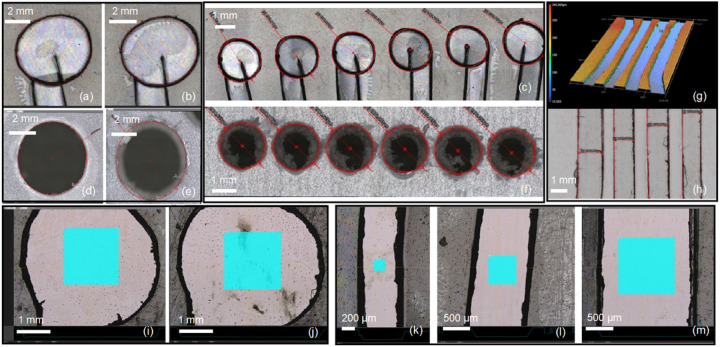
Physical characterization performed with laser confocal microscope on focus variation and laser rastering modes. (a-c) Double-side adhesive (DSA) ports processed with cutter plotter tool. Oval shaped ports were found to be produced for circular designs of 4 mm and 2 mm on 4-CH (a, b), and 6-CH devices (c) respectively. (d-f) Polycarbonate (PC) ports drilled with T2 and T4 milling bits on the micromilling tool for the 4-CH (d, e) and 6-CH devices (f). Due to tapered shape of the bits, inlet and outlet showed variation on its diameter as seen on d (wider) and e. Also, burr is observed for the 6-CH device (f) in the inner area of the ports. (g-h) Channel width characterization for cutter plotter processed DSA and its 3D representation on the 4-CH chip. (i-m) Surface roughness characterization on the Cr/Au layer for ports (i-j), and channels (k-m) at 200 μm^2^ (k), 500 μm^2^ (l) and 1000 μm^2^ (m) as evaluation areas, respectively. All data obtained from this characterization can be analysed in detail on the Supplementary Information (SI) tables S-2 to S-4

As observed in Fig. 3, microfluidic devices are composed by the inlet and outlet ports, and microchannels. These two features were fabricated using several materials and makerspace microfabrication tools. For the ports, Polycarbonate (PC) and Polymethyl Methacrylate (PMMA) were both successfully micromachined using micromilling and CO_2_ laser machining. On the other hand, double side adhesive (DSA) layers were cut using a precision cutter plotter tool in addition to the previous instruments used for the ports. Additional results of microchannel micromachining using Infrared (IR), Green and Ultraviolet (UV) laser sources are presented in the Table S-1. Ablation all the way through the DSA substrate was manifested for the Infrared (IR) and Green (GR) laser sources at high power, 100% output, 50 Hz repetition rate at 10 μm/s and 70 μm/s speed for one-side or double-side micromachining respectively.

#### 3.2.1 Process and device specification

As presented in Table S-2, optimized micromachining conditions and materials for the microfluidic chips are characterized and presented as a specification datasheets (spec sheets) showing the most relevant measured values during the fabrication processes along with the processing parameters for the four (4-CH) and six (6-CH) channels microfluidic chips that were used in this research.

**Figure 4:**
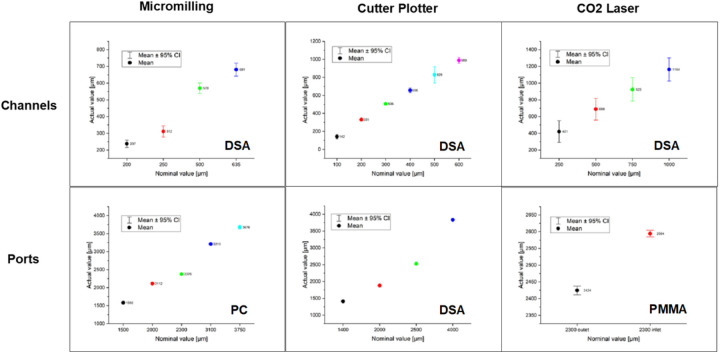
Variability on tools performance. Channels and ports features processed by three bulk micromachining technologies: micromilling, cutter plotter and laser, and same number of materials: double side adhesive (DSA), polycarbonate (PC) and Polymethyl Methacrylate (PMMA). PORTS: Lowest standard deviation on cutting was given by the CO2 laser, indicating a high precision. On the other hand, highest accuracy was obtained by the micromilling tool when used with specialized drilling bits. CHANNELS: “Air-Gap” micromilling was revealed to have the lowest standard deviation and fabrication error compared to CO2 laser and cutter plotter machining by producing accurate and precise channels vs design

Variability in the microchannel and inlet/outlet port dimensions using the different tools for DSA, PMMA and PC materials is presented in Fig. 4.

With a 95% of confidence interval in all the micromachining processes, the lowest standard deviation for the fabrication of 2.3 mm ports was obtained using the CO2 laser cutting having an average value of 13.6 μm, whereas for the micromilling tool with specialized drilling bit the same value measured 17.6 μm. In contrast, the lowest error was produced by the micromilling tool (2300 μm vs 2375.80 μm for on the design vs actual device measurement), which was lower than the CO2 laser (2300 μm vs 2509.36 μm average value measured for the inlet and outlet ports). This resulted in a fabrication error of 3.3% and 9.1% for the Micromilling and CO2 laser respectively. Additional statistics can be found in Table S-3. For the CO2 laser micromachining higher values for the inlet opening compared to outlet are observed due to higher energy of the laser beam striking the PMMA surface.

On the fabrication process for microchannels using DSA material, it was found that the highest standard deviation was obtained for the CO2 laser micromachining at 81.35 μm, and the lowest value for this parameter was 36.87 μm for the “Air-Gap micromilling” approach. The standard deviation using the cutter plotter was in between the other two methods at 72.47 μm. The error for the techniques for a 500 μm width microchannels were 13.96%, 37.7%, and 65.73% for “Air-Gap” micromilling, CO2 laser, and cutter plotter respectively.

#### 3.2.2 Surface roughness

For the optimal performance of MA, a flat surface is desired to enhance the chemical-to-mechanical energy exchange of the DNA motors (Piranej, Bazrafshan, and Salaita 2022), required for the example RA assay. An average surface roughness of 46.7 nm was obtained for the 6-CH device, measured at ports and channels over 3 different areas (200, 500 and 1000 μm2) which is excellent for the rolling of the 5 μm motor spheres (Fig. 3). For the 4-CH devices, surface roughness was found to be 20.5 nm obtained for 500 and 100 μm2 as representative areas on the metallized substrate. Materials, microfabrication tools, optimized process parameters, and additional specifications for the roughness characterization are further summarized in Table S-4.

### 3.3 Particle detection

**Figure 5:**
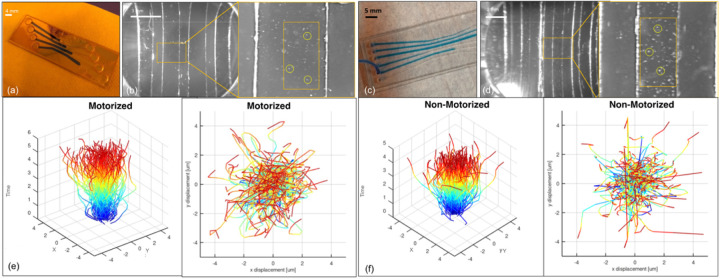
Microparticle detection and tracking of motion on the microfluidic chip. Microparticles detection and tracking on the microfluidic cartridges for 4-CH (a, b) and 6-CH devices (c, d). (b, d) Optical images taken from the ESP-32 imaging module tracking polystyrene microbeads (10 μm diameter) at a Brownian motion fashion, randomly selected for 4-CH and 6-CH chips, respectively. (e-f). Post-processing-colored plots produced by the RA system. “Medusa plots” generated for motorized (functionalized) on (e), and non-motorized (Brownian motion) beads depicted on (f), with translocation up to 4 μm along X and Y axes. Z axis is representing time, with the particle movement coming from blue to the red color

Visibility of 5 μm spheres was successfully demonstrated utilizing the wireless optical kit when implemented on both 4-CH and 6-CH chips. Fig. 5(a–d) depicts beads in the 4-CH and 6-CH chips, respectively. Microbeads were identified as bright spots in the obtained images. The diameters of microbeads ranged from 4 to 6 pixels, based on background brightness and overall contrast. Results for sub-pixel motion tracking in an X/Y coordinate system representing the microchannel surface are additionally shown in Fig. 5(e–f) for motorized and non-motorized particles. In this “Medusa plot”, time is represented on the z-axis as well as color (blue = start and red = end of tracking). Initial data analysis of a subsection of the entire frame detected 556 potential 5 μm motor spheres and the motion of 227 spheres was successfully tracked for up to 15 minutes. Each microparticle’s position was normalized and the displacement plotted in superposition. The test-spheres traveled between 1 and 4 μm under the influence of Brownian motion.

#### 3.3.1 Non-Motorized microspheres

Concentrations of polystyrene microbeads (10 μm diameter; 10μL/1mL and 20μL/10mL in DI water) were initially pushed into the microchannel by applying pressure in the inlet port and then they were driven by Brownian motion for approximately 5 minutes. The ESP-32 CAM optical system took 16 pictures to track 219 beads. Side-by-side comparison of the ESP-32 CAM optical system with a standard transmitted light optical microscope (Nikon, Eclipse TS2), at 20x magnification can be visualized in Fig. S-1. Individual spheres are easily visualized in the microscope images while it is harder to visualize them in the Wi-Fi CAM module but motion tracking of sets of spheres used in the MA system are possible in the Wi-Fi CAM module.

#### 3.3.2 Motorized microspheres

Conversely, DNA-functionalized silica beads (5 μm diameter) motors were created for rolling onto the RNA-functionalized substrate required for the RA. Aptamer hybridization followed by the addition of the fuel (i.e., RNase) to cause the rolling motion of the motors by degrading the DNA-RNA complex (hydrolysis) for 15 minutes allowed to take 45 pictures for further processing and successful tracking of 117 beads.

In the case where the number of rolling spheres to be detected is increased, the processing time is proportional to that variable, and a large visual field would be necessary, especially for non-motorized microspheres.

### 3.4 Virus detection

Virus detection and DNA motors testing was carried out experimentally with actual COVID-19 virus (Delta variant) as depicted in Fig. S-2.

Using a PMMA/DSA-based 4-CH device allowed to successfully detect 10^7^ copies/mL of COVID-19 virus. Timelapse screenshots each 5 seconds are presented in Fig. S-2 (green and blue rectangles) where it is easily observable that in the absence/presence of virus in the sample, the RA is highly effective. The assay prevents DNA motors from rolling along the microfluidic chips indicating a positive result, i.e., COVID-19 infection (no depletion tracks). On the other case, when no virus is present on the sample, the motors roll freely by consuming the RNase reagent leaving a depleted track (black color) in the fluorescence images, indicating a negative result, i.e., no COVID-19 infection. This detection was performed implementing the assay in its originally conceived setup using specialized microscopy. The results at different virus concentrations (including COVID-19 Delta variant among them) are currently in press, work developed by Salaita Lab in Emory University. Translation to a different strain of virus and running a parallel scan on the same microfluidic chip can be obtained by introducing functionalized DNA motors with the specific aptamer for the virus/strain of interest (not showcased in this paper).

### 3.5 Microfluidic device modelling

#### 3.5.1 Flow rate, share stress and pressure modelling

Evaluation of several mechanical aspects of the PC and PMMA materials along the microchannel structure such as flow rate, share stress and pressure were modelled to obtain the limits of operation of the microfluidics devices as depicted in Fig. 5(a–d). Two limiting flow rates were modelled at 50 μL/min and 25 mL/min respectively. These results were later corroborated to actual characterization of leaking and flow rate measurement on actual microfluidic cartridges as presented in Table S-5. Modelling showed that microchannels on average can withstand 0.077 and 38 Pa (Shear Stress), 8 and 4411 Pa (Relative Pressure) along the top surface of PC and PMMA simulated at 50 μL/min, and 25 mL/min flow rate respectively. Similarly, the simulation provided average values of 0.02 and 11.95 Pa (Shear Stress) along the DSA surfaces representing the side faces of the microchannel structure (Table S-6). Taking advantage of the setup for the flow rate testing, characterization of leak performance of the chips was obtained pushing the microchannels to its flow limits. Automatic flow rates of 50 and 1000 μL/min, and 2, 5, 10 and 25 mL/min were injected into the microfluidic chip. The experimental volume per microchannel was 12 μL, and 9 μL for PMMA and PC respectively. PMMA and DSA showed an excellent bonding strength as no leaking of the flowing fluid was observed at any of the flow rates. On the other case, PC showed significant leaking at flow rates beyond 5 mL/min imposing operation limits when this material was implemented as top layer of the microchannel.

### 3.6 UV-Vis spectroscopy

Transmittance evaluated in the entire spectra from 200 nm to 1000 nm with special focus on the visible range from 400 to 800 nm was obtained for all the layers in the microfluidic cartridge. Clear PMMA, PC and Glass were used as references and compared to functionalized surfaces when reagent bis-TPA was added. The reagent was air- and oven-dried at 0.1%, 1%, and 2% concentrations to analyze hydrophilicity improvement as depicted in Fig. 5(e–h). Both materials (i.e. PMMA and PC) in an unprocessed state showed excellent transmittance in the visible spectra with average values of 92.33% and 91.94% respectively, similar to those reported by Shih et al (Shih et al. 2013). The “sinusoidal” transmittance spectrum of PC is comparable to previous studies and is attributable to the internal losses in different frequencies of light in the native material.(Polycarbonate 2010) Resin structure and quality, regrind usage, and methods of manufacturing are among the reasons for these internal losses. When comparing methods of bis-TPA drying (i.e., air and oven), they both showed a worsening trend for transmittance on both PC and PMMA surfaces coated at 2% Bis-TPA. Higher losses were found on the PMMA material for 1% and 2% Bis-TPA concentration at approximately 92% for the oven drying setup. On the bottom most layer of microfluidic cartridge (metallized glass with 3nm_Cr and 5nm_Au), the transmittance was well below the Glass reference (average value of 54.43%) in the visible spectrum. While bis-TPA did not impact the transmission of light through the inlet/outlet materials, as expected the biggest drop was found to be for the metallized glass. While sophisticated microscopes such as Eclipse Ti2-E Nikon are able to overcome this drop in transmittance of the microfluidic cartridges adequately to perform MA, the Wi-Fi imaging kit tracks the microparticles from a bottom to top projection transmission of light. To overcome the transmittance reduction due to the metallization process of the glass substrate, supporting LEDs are integrated to the 3D printed housing of the system enhancing the tracking of particles and functionalized motors.

### 3.7 Contact angle measurements (CAM)

**Figure 7:**
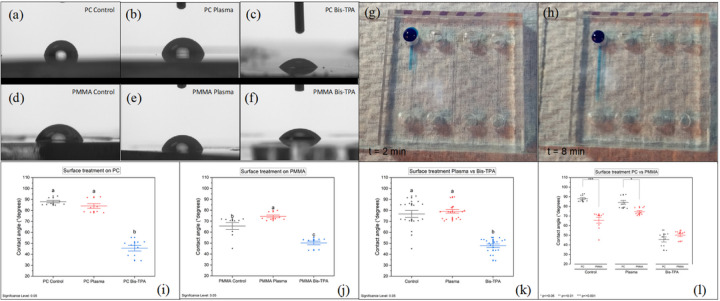
Wet contact angle measurements (CAM) on PC and PMMA. Single event measurements to compare O_2_ plasma treatment and Bis-TPA reagent for hydrophilicity studies are illustrated on (a-c) for PC and (d-f) for PMMA. It was revealed the positive impact on the reduction of the angle (increased hydrophilicity) when the material is Bis-TPA treated, going from 88° in control to 46° in the PC treated surface. The same tendency was observed for PMMA surface going down from 67° to 50°. (g-h) exemplifies the hydrophilicity test on actual cartridges at two different moments. Passive flowing of Methylene blue inside the microchannels after 2 min and 8 min is depicted. It is observed that a distance (around 80% of the channel length) is covered without applying any pressure on the inlet port and no gravity effect is either involved. (i-l) Multievent statistics analysis on both materials (PC and PMMA). Bis-TPA treatment (blue scattering) is showing more hydrophilicity effectiveness than O_2_ plasma treatment (red scattering) on each polymer, resulting in lower contact angle values

Single- and multi-event contact angle measurements were carried out on PC and PMMA materials as depicted in Fig. 7, with results suggesting enhancement in the flow properties along the microchannels. Bis-TPA reagent was used to improve hydrophilicity along the inner surfaces of the cartridge microchannels to enhance the flow of reagents that are associated with microbeads-based and virus assays. Surface functionalization at 1% V/V Bis-TPA/Ethanol concentration provided improved reduction of the contact angle and hence enhanced hydrophilicity when compared to plasma treated surfaces on standard Left-Right and Young-Laplace CAM as presented in Table S-7 for a single-event measurement. On the case of PC samples (Control, Plasma treated, and Bis-TPA functionalized), they presented 87.6°, 84.3°, and 45.8° CAM respectively. Alternately, PMMA samples showed 67.1°, 74°, and 50.2° on the same metrics showcasing effectiveness of the Bis-TPA layer in improvement of surface hydrophilicity. Interestingly, PMMA didn’t show any improvement after surface plasma treatment.

Multi-event CAM taken at one-week intervals for 5 weeks indicated that functionalization at 2% V/V Bis-TPA/Ethanol concentration onto PMMA surfaces works better when samples are oven dried overnight @ 60 °C compared to ambient air drying and Young-Laplace CAM method is implemented accounting for surface tension forces as plotted in Fig. S-3. These results are consistent with increase in contact angle (CA) directly proportional to the time indicating reduction of the initial hydrophilicity (Table S-8).

## 4. Conclusions

The conclusions for this project might be split in three main aspects: microfabrication process development, optical processing, and particle/virus detection.

First, laser micromachining, 3D printing, micromilling, and precise plotter cutting were evaluated to optimize the microfabrication process for a disposable microfluidic chip. This represents a major step forward the multiplexing capability for detection of several strains of SARS-CoV-2 and paves the way to address upcoming challenges with new variants. As the core kit functionality does not depend on the analyzed virus, one-step reagent modification might take place on the surface and motors functionalization to switch from one virus to another.

“Air-Gap” micromilling provided the lowest standard deviation for microchannel dimension variation compared to other methods on DSA followed by cutter plotter and laser tools. Errors and differences between the design and the actual device dimensions were mostly due to the manual steps during the assembly process for the multilayered chip. During the microfabrication of ports on PC and PMMA, the lowest deviation from the design parameters was provided by the CO_2_ laser and micromilling provided large standard deviations but accurate port dimensions. Interpretation of this results represents high precision for the CO_2_ laser and the high accuracy for the micromilling tool for material micromachining. Cutter plotter tool was evaluated for the processing of circular shapes (i.e., ports) using DSA as material. It was interesting to see this feature resulted in very low deviation error, but a noticeable change in its shape was obtained resulting in oval ports instead of circular ones.

Flow rate study provided information about on quality of the bonding between DSA/PMMA and DSA/PC. PMMA showed to have higher adherence to the DSA with no evident leaking up to 25 mL/min. PC bonded chips leaked at 5mL/min and above. This might be attributed to the surface incompatibility between the silicone-based adhesive used as the microchannels structure and the polymers. Improvements to flowing liquid along the microchannels were obtained after Bis-TPA surface functionalization on both polymer surfaces.

Second, the Wi-Fi imaging module provided accurate detection of microparticles in the sub-pixel motion range along x-y plane, and versatility to access results instantaneously from any portable device with wireless capability such as mobile phones, tablets, or laptops. Generation of colored plots for particle motion, direction, and speed were obtained allowing the individual identification of functionalized motors for virus detection and non-functionalized beads for particle tracking. Also, algorithm optimization was performed by implementing numerical methods for invalid tracks removal, drift correction, image noise reduction, and tracks smoothening. UV-Vis spectroscopy provided a challenge during the implementation of an transmitted light microscopy approach for laboratory tests. However, increasing the power of light by using laterally placed white LEDs will overcome this issue allowing the system to detect and track DNA-motors and microbeads reliably.

Lastly, the optical kit along the microfluidic chip successfully detected microparticles in the range of 5–10 μm size under the influence of the RA (DNA motors) and Brownian motion (Polystyrene and Silica beads). Most importantly, the device was successfully tested with actual viruses (COVID-19, Delta Strain) providing a proof-of-concept detection of 10^7^ copies/mL. These results could be potentially automatically uploaded by the system to the cloud and accessed by the customer from any place via text message, email or push notification.

## Supplementary Material

Supplement 1

## Figures and Tables

**Figure 1:**
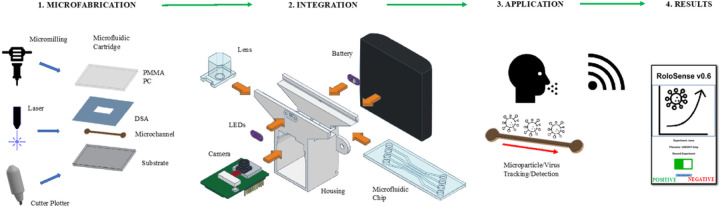
Schematic process for the system development: 1. Microfabrication approaches for rapid prototyping of multilayered chip on a variety of materials, 2. Integration of microfluidic chip with optical imaging kit in exploded view (camera, LEDs, 3D printed housing, microfluidic chip, battery holder, and lens), 3. Proof-of-concept application for virus detection (COVID-19 Delta variant), and microparticle tracking (Motorized and Non-motorized), and 4. Report of results via Wi-Fi on mobile device app

**Figure 6:**
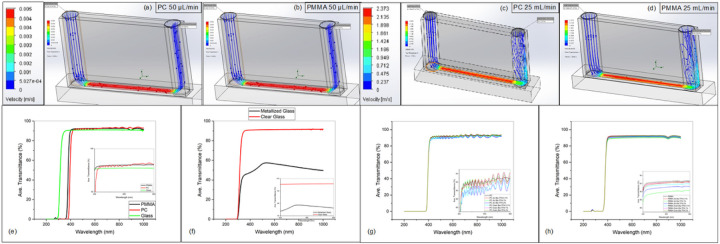
Fluid dynamics simulation (Flow rate, shear stress and pressure) and UV-Vis characterization. PC and PMMA microchannels are exposed to 50 μL/min (a, b) and 25 mL/min (c, d) flow rate. Velocity profile is presented on the enlarged color bar next to each flow rate test. UV-Vis characterization of plain and Bis-TPA functionalized polymers, and clear and metallized glass substrate. (e) Transmittance (T) spectrum on the range from 200 nm to 1100 nm with inset on the visible range from 400 nm to 800 nm. Extraordinary transparency is obtained for polymers, even greater than clear glass is noted on the visible range. All materials presented T > 90% making them ideal for upright microscopy applications. (f) Transmittance comparison between clear and metallized (3 nm Cr/5 nm Au) glass substrate for RA. Average value of 54% T for the metallized surface is exhibited on the inset. (g) Transmittance spectrum for functionalized PC. Slight increase on the averaged T from 91% to 91.2% for both drying methods at different concentrations (i.e., 0.1%, 1%, and 2% V/V) is displayed. Inset reveals decreasing T for 0.1% Bis-TPA (red-air and cyan-oven lines) and increasing T for 2% Bis-TPA (blue-air and brown-oven lines) when comparing from air drying to oven drying technique results. Wavy shape on the spectrum is usually gotten for variations on methods of production and raw materials used for the PC sheet fabrication. (h) Transmittance spectrum for functionalized PMMA. Bis-TPA coating on the oven drying method presented better T values on average than air drying. Air dried Bis-TPA at 1% and 2% fall below 91% on the visible range as observed on the green and blue lines
