# Supplementary material for: High-throughput microbead assay system with a portable, cost-effective Wi-Fi imaging module, and disposable multi-layered microfluidic cartridges for virus and microparticle detection, and tracking
Source: Res Sq. 2022 Dec 20:rs.3.rs-2383455. Preprint. [Version 1] doi: 10.21203/rs.3.rs-2383455/v1 (PMC9810214; doi:10.21203/rs.3.rs-2383455/v1)
Supplement: Supplement 1 [file NIHPPRS2383455v1-supplement-1.pdf]

Supplementary Files

This is a list of supplementary files associated with this preprint. Click to download.

- SIFINAL.pdf
